# Overweight and obesity as predictors of early mortality in Mexican children with acute lymphoblastic leukemia: a multicenter cohort study

**DOI:** 10.1186/s12885-019-5878-8

**Published:** 2019-07-18

**Authors:** Juan Carlos Núñez-Enríquez, Ana Elena Gil-Hernández, Elva Jiménez-Hernández, Arturo Fajardo-Gutiérrez, Aurora Medina-Sansón, Janet Flores-Lujano, Laura Eugenia Espinoza-Hernández, David Aldebarán Duarte-Rodríguez, Raquel Amador-Sánchez, José Gabriel Peñaloza-González, José Refugio Torres-Nava, Rosa Martha Espinosa-Elizondo, Luz Victoria Flores-Villegas, Laura Elizabeth Merino-Pasaye, María Luisa Pérez-Saldivar, Elisa María Dorantes-Acosta, Beatriz Cortés-Herrera, Karina Anastacia Solis-Labastida, Nora Nancy Núñez-Villegas, Martha Margarita Velázquez-Aviña, Angélica Rangel-López, Ana Itamar González-Ávila, Jessica Denisse Santillán-Juárez, Alejandra Jimena García-Velázquez, Silvia Jiménez-Morales, Vilma Carolina Bekker-Méndez, Haydee Rosas-Vargas, Minerva Mata-Rocha, Omar Alejandro Sepúlveda-Robles, Jorge Alfonso Martín-Trejo, Juan Manuel Mejía-Aranguré

**Affiliations:** 1 Unidad de Investigación Médica en Epidemiologia Clínica, UMAE Hospital de Pediatría “Dr. Silvestre Frenk Freund”, Centro Médico Nacional “Siglo XXI”, Instituto Mexicano del Seguro Social (IMSS), Mexico City, Mexico; 2 Servicio de Hematología Pediátrica, Hospital General “Gaudencio González Garza”, Centro Médico Nacional “La Raza”, IMSS, Mexico City, Mexico; 3 Servicio de Hemato-Oncologia, Hospital Infantil de México Federico Gómez, Secretaria de Salud (SS), Mexico City, Mexico; 4 Servicio de Hematología Pediátrica, Hospital General Regional “Carlos McGregor Sánchez Navarro”, IMSS, Mexico City, Mexico; 5 Servicio de Onco-Pediatria, Hospital Juárez de México, SS, Mexico City, Mexico; 6 Servicio de Oncología, Hospital Pediátrico de Moctezuma, Secretaria de Salud de la Ciudad de México (SSCDMX), Mexico City, Mexico; 7 Servicio de Hematología Pediátrica, Hospital General de México, SS, Mexico City, Mexico; 8 Servicio de Hematología Pediátrica, Centro Médico Nacional “20 de Noviembre”, Instituto de Seguridad y Servicios Sociales de los Trabajadores del Estado (ISSSTE), Mexico City, Mexico; 9 Servicio de Hematología Pediátrica UMAE Hospital de Pediatría “Dr. Silvestre Frenk Freund”, Centro Médico Nacional “Siglo XXI”, IMSS, Mexico City, Mexico; 10 Coordinación de Investigación en Salud, IMSS, Mexico City, Mexico; 11 Servicio de Hemato-oncología Pediátrica, Hospital Regional No. 1° de Octubre, ISSSTE, Mexico City, Mexico; 12 Laboratorio de Genómica del Cáncer, Instituto Nacional de Medicina Genómica (INMEGEN), Mexico City, Mexico; 13 Unidad de Investigación Médica en Inmunología e Infectología, Hospital de Infectología “Dr. Daniel Méndez Hernández”, “La Raza”, IMSS, Mexico City, Mexico; 14 Unidad de Investigación en Genética Humana, UMAE Hospital de Pediatría “Dr. Silvestre Frenk Freund”, Centro Médico Nacional “Siglo XXI”, IMSS, Mexico City, Mexico

**Keywords:** Children, Leukemia, Overweight, Obesity, Early mortality, Mexico

## Abstract

**Background:**

Mexico City has one of the highest incidences and mortality rates of acute lymphoblastic leukemia (ALL) in the world and a high frequency of early relapses (17%) and early mortality (15%). Otherwise, childhood overweight and obesity are reaching epidemic proportions. They have been associated with poor outcomes in children with ALL. The aim of present study was to identify if overweight and obesity are predictors of early mortality and relapse in Mexican children with ALL.

**Methods:**

A multicenter cohort study was conducted. ALL children younger than 15 years old were included and followed-up during the first 24 months after diagnosis. Overweight and obesity were classified according World Health Organization (WHO) and Centers for Disease Control and Prevention (CDC) criteria. Early mortality and early relapses were the main outcomes.

**Results:**

A total of 1070 children were analyzed. Overweight/obesity at diagnosis were predictors of early mortality (WHO: HR = 1.4, 95%CI:1.0–2.0; CDC: HR = 1.6, 95%CI:1.1–2.3). However, no associations between overweight (WHO: HR = 1.5, 95%CI:0.9–2.5; CDC: HR = 1.0; 95% CI:0.6–1.6) and obesity (WHO: HR = 1.5, 95%CI:0.7–3.2; CDC: HR = 1.4; 95%CI:0.9–2.3) with early relapse were observed.

**Conclusions:**

Overweight and obese patients embody a subgroup with high risk of dying during leukemia treatment.

**Electronic supplementary material:**

The online version of this article (10.1186/s12885-019-5878-8) contains supplementary material, which is available to authorized users.

## Background

Mexico has one of the highest mortality rates of childhood acute lymphoblastic leukemia (ALL) worldwide [1]. In spite of using the same chemotherapy schemes as those used in developed countries, mortality has been increasing in recent years [2], mainly in the first stages of treatment [3].

Mortality during the induction remission phase in developed countries is ~ 1–2% [4, 5]. Nevertheless, in developing countries, mortality during this treatment stage is extraordinarily high as it has been reported in Honduras (20.8%) [6], Brazil (14.9%) [7], and India (17%) [8]. In Mexico City, Rivera Luna et al [9] also reported a high mortality rate (15%) during induction stage. Furthermore, it has been observed that early relapses are amongst the main obstacles to achieve better ALL survival rates in Mexican children; they occur in a higher (17–22.1%) [3, 10] proportion than in developed countries (3–4.5%) [11].

Among the factors that could impact on childhood ALL prognosis, nutritional status has been investigated [12]. Importantly, overweight and obesity at the time of diagnosis have been associated to a high risk of relapse and death in children with ALL [3, 13, 14].

There are hypotheses that could explain the susceptibility to relapse and chemotherapy drug resistance in overweight and obese patients. In this regard, it has been pointed out, that adipocytes encapsulate leukemic cells conferring them resistance to chemotherapy drugs in a protected microenvironment [15].

Up to this moment, several international studies have been performed to assess the association between overweight and obesity with relapse and survival rates in ALL children [14, 16–19]. Body mass index (BMI) and its classification according to the CDC and WHO nutritional charts for age and sex has been widely used to evaluate these nutritional alterations [13, 20]. Notwithstanding, a consensus among authors on the impact of BMI in pharmacokinetics, toxicity and chemotherapy effectiveness has not been reached yet [21].

Taking into consideration that in Mexico the prevalence of overweight and obesity, and mortality rates of childhood ALL are high and have been increasing in the last years [1, 22–25], and also considering that nutritional status is a potentially modifiable prognostic factor, the aim of the present study was to evaluate if overweight and obesity are associated with a high risk of early relapse and mortality in our population.

## Methods

### Participants

The Mexican Interinstitutional Group for the Identification of the Causes of Childhood Leukemia (MIGICCL) conducted a multicenter retrospective cohort study in eight public hospitals of Mexico City. Children diagnosed with ALL between January 1st 2010 and December 31st 2013 at any of participant hospitals were included. Down syndrome children and Mexico City non-resident patients were excluded. A follow-up of 24-months was performed to each child from the moment of diagnosis confirmation. Diagnosis of ALL was based on the morphologic and immunophenotypic features of leukemic cells.

Participant Institutions were *Instituto Mexicano del Seguro Social* (IMSS), *Secretaría de Salud* (SS), and *Secretaría de Salud de la Ciudad de México*; and *Instituto de Seguridad y Servicios Sociales de los Trabajadores del Estado* (ISSSTE). All patients were treated according to the chemotherapy protocol used in the hospital where they received medical care.

### Data collection

Information regarding sex, age at diagnosis, place of residence, white blood cell (WBC) count, immunophenotype (B or T lineage), weight and height (length when appropriate) at diagnosis, and chemotherapy protocol was collected from the patients’ clinical charts by previously standardized staff. Overcrowding was used as a proxy for socioeconomic status (SES) according to the Bronfman’s criteria (high SES, up to 1.5 people per room; medium–low SES, more than 1.6 people per room) [26]. The risk classification was according to the criteria of the National Cancer Institute (NCI): standard risk [ages from 1 to 9.99 years; WBC count < 50 × 10^9^/L] or as high risk [age < 1 or ≥ 10 years or WBC ≥ 50 × 10^9^/L]. In the present study, early mortality was defined as a patient’s death at any moment during the first 24-months after diagnosis confirmation. Early relapse in bone marrow was defined when a patient presented ≥25% lymphoblasts in a bone marrow aspirate after complete remission (CR) achievement. Central nervous system (CNS) relapse was characterized as the presence of morphologically identified lymphoblasts on smears of cerebrospinal fluid (CSF) cytocentrifuge preparations with a mononuclear cell count ≥5/ml or cranial nerve paralysis, following the first CR.

### Assessment of nutritional status at diagnosis of ALL

BMI at diagnosis was used in the analysis. Using WHO Anthro and AnthroPlus for PC software (version 3.2.2, World Health Organization, Geneva), the BMI-for-age Z-scores were calculated for each patient. According to WHO classification, patients were categorized as normal (− 1.9999 to 0.9999), wasted (− 2 to − 2.9999), severely wasted (≥ − 3), at risk of overweight (1–1.9999), overweight (2 to 2.9999) and obesity (≥3) [27]. In addition, the BMI percentiles cutoffs provided by CDC were: normal (p5–84.9999), underweight (< p5), overweight (p85–94.9999), and obese (≥ p95). The nutritional classification and measurements validation regarding weight and height recorded in clinical files used to classify patients’ nutritional status in present research has been previously described [3]. Underweight patients were excluded from the analyses.

### Statistical analysis

Data analyses were performed using SPSS, version 21 (IBM Corp). Descriptive statistics and relative risks (RR) calculation with 95% confidence intervals (CI) were carried out. Kaplan–Meier survival analysis was carried out for early relapse and early mortality. Log-rank test was calculated. The analyses were conducted independently for early relapse or early mortality, adjusting for variables whose effects on the studied outcomes have been previously documented (age, sex, SES, immunophenotype, NCI risk classification, and chemotherapy protocol).

A high correlation (0.74) using correlation matrix analysis was observed between age and NCI risk classification. Therefore, it was decided to eliminate the variable age from the model. No interactions were identified. As a result, the most parsimonious model included sex, SES, immunophenotype, NCI risk classification, chemotherapy protocol and nutritional status. A Cox proportional hazard model was used. Hazard ratios (HR) with 95% CIs were calculated. In addition, Cox regression analyses stratified by age groups and NCI risk classification were performed adjusting for the variables included in the model.

## Results

During study period, a total of 1254 children were diagnosed with ALL in participating hospitals. Of these, 26 (2.1%) were Down syndrome patients, 113 (9%) could not be followed-up because they were Mexico City non-residents, and 45 children whose information regarding weight and height was not found in clinical charts. In total, 1070 (85.3%) patients met all selection criteria and were analyzed (Table 1).

**Table 1:** Clinical characteristics of ALL patients diagnosed between 2010 and 2013 in participating public hospitals of Mexico City

Variables	Total population	Analyzed		*p* ^a^
		yes	no	
	n (%)	n (%)	n (%)	
	1254 (100)	1070 (85.3)	184 (14.7)	
Sex				
Male	684 (54.5)	595 (55.6)	89 (48.4)	0.07
Female	570 (45.5)	475 (44.4)	95 (51.6)	
Age (years)				
< 1	34 (2.7)	28 (2.6)	6 (3.3)	0.29
1–9.9	840 (67.0)	726 (67.9)	114 (62.0)	
≥ 10	380 (30.3)	316 (29.5)	64 (38.8)	
Socioeconomic status				
Medium-low	1043 (85.2)	902 (85.7)	141 (82.0)	0.20
High	181 (14.8)	150 (14.3)	31 (18.0)	
WBC count at diagnosis (×10^9^/L)				
< 10	605 (48.2)	526 (49.2)	79 (42.9)	0.18
10–49.99	373 (29.7)	306 (28.6)	67 (36.4)	
50–99.99	107 (8.5)	91 (8.5)	16 (8.7)	
≥ 100	169 (13.5)	147 (13.7)	22 (12.0)	
NCI risk classification				
Standard	691 (55.1)	597 (55.8)	94 (51.1)	0.24
High	563 (44.9)	473 (44.2)	90 (48.9)	
Immunophenotype				
Pre-B	1077 (85.9)	915 (85.5)	162 (88.0)	0.60
B mature	20 (1.6)	16 (1.5)	4 (2.2)	
Biphenotypic	42 (3.3)	37 (3.5)	5 (2.7)	
T Cell	115 (9.2)	102 (9.5)	13 (7.1)	

^a^Pearson chi-square

### Classification of patient’s nutritional status

In accordance with WHO classification 61.5% (*n* = 658) had a normal weight, 18.4% were at risk of overweight (*n* = 197), 8.9% (*n* = 95) were classified as overweight and 4.2% (*n* = 45) as obese. With CDC classification, 13% (*n* = 129) were classified with overweight, and 14.1% (*n* = 140) as obese. Most of the patients classified as overweight by the WHO (92%), also fulfilled criteria for obesity according to the CDC classification (Additional file 1: Table S1).

A total of 160 (15%) early relapses occurred. Thirty-five (21.9%) of them befell during the first 6 months, and 20 (12.5%) between 18th and 24th month. The main sites of relapse were bone marrow (66.9%; *n* = 107), followed by isolated CNS relapse (*n* = 32; 20%). Early mortality frequency during the first 24 months after diagnosis was 19.9% (*n* = 213). Of these, 148 (69.5%) occurred during the first year of treatment. Main causes of death were: septic shock (*n* = 141; 66.4%), hemorrhagic shock (*n* = 30; 13.9%), and leukemic activity (*n* = 24; 11.3%). An increased risk of early relapse and death in children < 1 and ≥ 10 years of age was observed (Table 2).

**Table 2:** Bivariate analysis, very early relapse and early mortality during the first 24 months after diagnosis

Variable	Very early relapse			Early mortality		
	# events	RR	IC 95%	# events	RR	IC 95%
Sex						
Female (ref.)	54	1	–	103	1	–
Male	106	1.7	1.2–2.4	110	0.8	0.6–1.1
Age						
1–9.9 years (ref.)	92	1	–	111	1	–
< 1 year	8	2.7	1.2–6.4	15	6.4	2.9–13.8
≥ 10 years	60	1.6	1.1–2.3	87	2.1	1.5–2.9
NCI risk classification						
Standard (ref.)	66	1	–	81	1	–
High	94	1.9	1.4–2.8	132	2.5	1.8–3.3
Immunophenotype						
Pre-B (ref.)	138	1	–	173	1	–
B mature	4	1.8	0.6–5.9	3	0.9	0.3–3.5
Biphenotypic	5	0.8	0.3–2.3	7	1.0	0.4–2.3
T Cell	13	0.8	0.4–1.5	30	1.8	1.1–2.8
Socioeconomic Status						
High (ref.)	32	1	–	32	1	–
Low-medium	127	0.6	0.4–0.9	173	0.9	0.6–1.3
Nutritional status						
CDC (percentiles)						
Normal (p5–84.9) (ref.)	79	1	–	95	1	–
Overweight (p85–94.9)	19	1.1	0.6–1.9	29	1.5	0.9–2.4
Obesity (≥ p95)	25	1.4	0.8–2.2	39	1.9	1.3–3.0
WHO (Z-score)						
Normal (−1.9–0.9) (ref.)	96	1	–	118	1	–
At risk of overweight (1–1.9)	28	0.9	0.6–1.5	45	1.4	0.9–2.0
Overweight (2–2.9)	18	1.4	0.8–2.4	25	1.6	1.0–2.7
Obesity (≥3)	8	1.3	0.6–2.8	12	1.7	0.8–3.3

*ref* reference category, *RR* relative risk, *95% CI* 95% confidence interval

### Using CDC nutritional classification

**Fig. 1:**
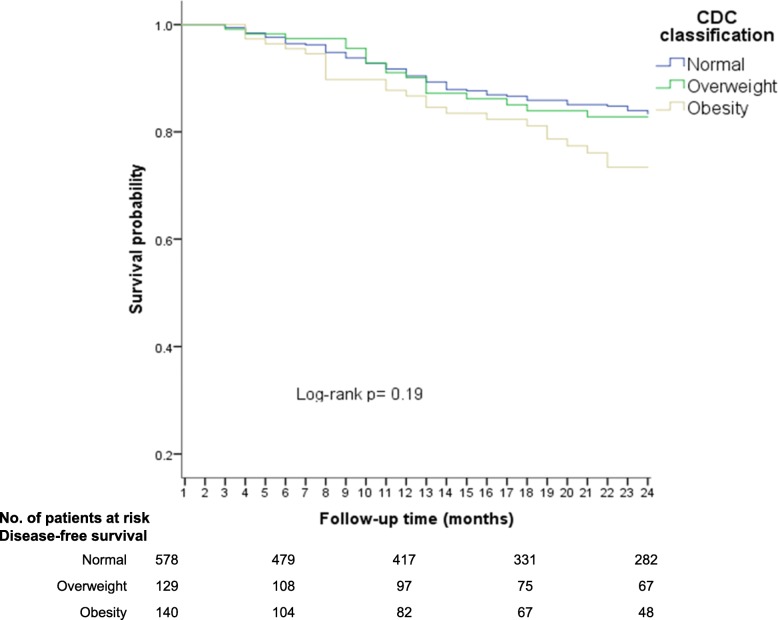
Disease-free survival (early relapses) according to CDC 2000 nutritional classification (three categories)

**Fig. 2:**
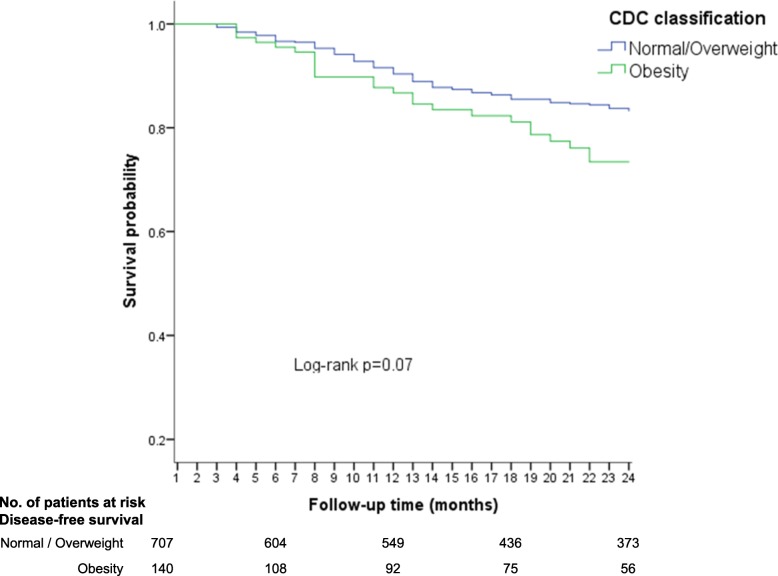
Disease-free-survival (early relapses) according to CDC 2000 (two categories)

The lowest disease free-survival (DFS) rates during follow-up time (24 months) were observed in children with obesity (73%), in contrast with the normal weight (81%; *p* = 0.07) and overweight (80%; *p* = 0.20) groups (Fig. 1). When the normal weight/ overweight groups were used as the reference category, the obesity group had the lower DFS, nonetheless, a low precision was noted (Log-rank; p = 0.07) (Fig. 2).

In Cox regression analysis, no association was observed between overweight and early relapse (HR = 1.0; 95% CI: 0.6–1.6); however, a high risk of relapse was noted in the obese group, eventhough the confidence intervals were imprecise (HR = 1.4; 95% CI: 0.9–2.3).

**Fig. 3:**
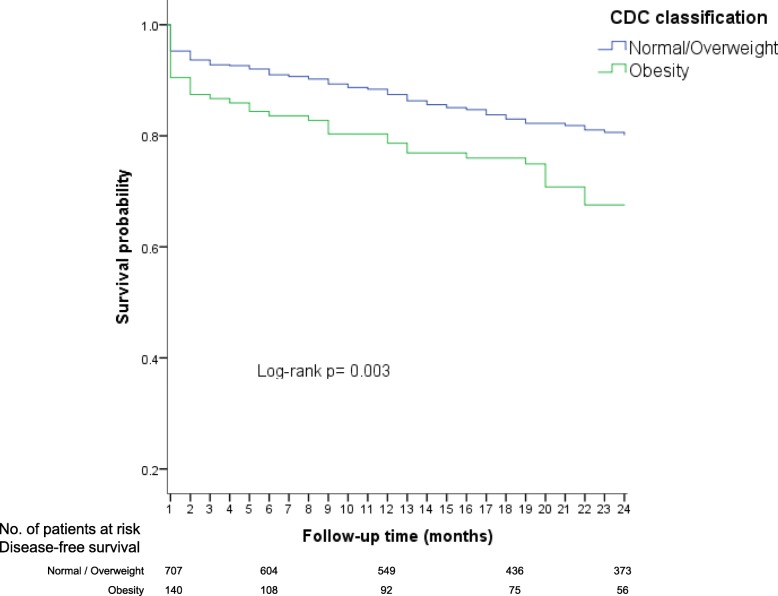
Overall survival up to 24 months according to CDC nutritional status

Likewise, a lower overall survival (OS) up to the first 24 months was observed in children in the obesity group (68%), whereas patients with overweight had a slightly greater OS (75%), but not as much as patients with a normal weight (81%). When, patients with normal weight and overweight were categorized together (as the reference group), and compared with obese children, differences in survival were more noticeable (Log-rank; *p* = 0.003) (Fig. 3).

In the Cox regression analysis, an association between obesity and an early mortality increased risk was observed (HR = 1.6, 95% CI: 1.1–2.4). Still, for risk estimation between overweight and early mortality the confidence intervals were not precise (HR = 1.2, 95% CI: 0.7–1.8). Then, the normal weight and overweight patients (as the reference group) were grouped in the same category, and afterwards, compared with the obese group. Results confirmed an increased risk of early mortality in patients with obesity (HR = 1.6, 95% CI: 1.1–2.3).

In a separate analysis, the association between obesity and early mortality also was assessed stratifying by NCI risk classification considering the normal weight and overweight children as the reference category. Similar results were observed in the subgroup of standard-risk (HR = 1.6, 95% CI: 0.8–3.1) and high-risk patients (HR = 1.7, 95% CI: 1.1–2.7).

In another analysis stratified by age, the risk of early mortality in the group of obese patients was higher in the age group ≥10 years (HR = 2.1, 95% CI: 1.3–3.5) than in the group of 1–9.9 years (HR = 1.2, 95% CI: 0.6–2.2).

### Using the WHO nutritional classification

**Fig. 4:**
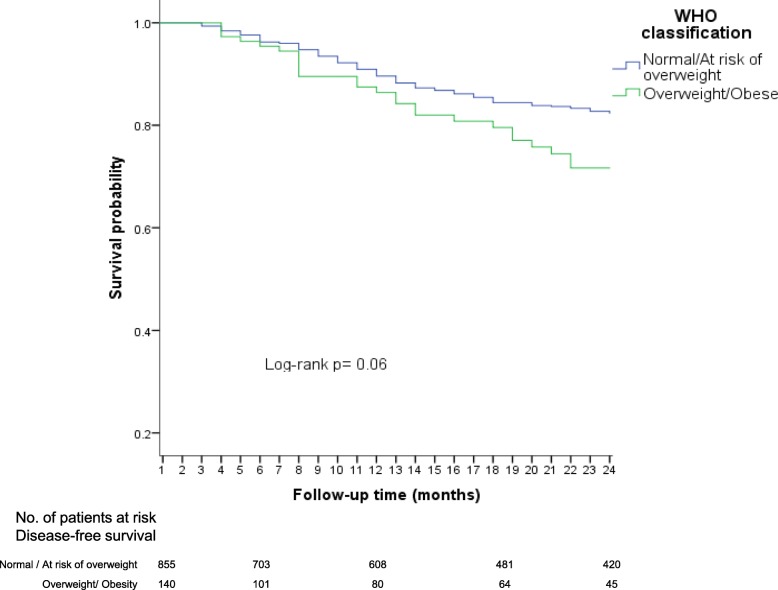
Disease free-survival (early relapses) according to WHO nutritional classification

The major DFS rates were observed for children with normal weight and those at risk of overweight, with 80 and 81%, respectively. In contrast, the lower DFS rates were observed in overweight (71%) and obesity children (73%). Afterwards, we grouped in a same category those patients with normal weight or at risk of overweight (as the reference category) and compared them to the overweight/obesity category. DFS was lower in the overweight /obesity group (72%) compared with the reference group (81%), nevertheless, a low precision was observed (Log-rank; *p* = 0.06) (Fig. 4).

In the Cox model, the results exhibited that overweight and obesity were associated with an increased risk of early relapse, but confidence intervals were imprecise (HR = 1.5, 95% CI: 0.9–2.5 and HR = 1.5, 95% CI: 0.7–3.2; respectively). Then, when a separated analysis was carried out, considering as the category of reference patients with normal weight and at risk of overweight, and in another category those children with overweight and obesity, an association with early relapse was evidenced. Nevertheless, the confidence interval was imprecise (HR = 1.4, 95% CI: 0.9–2.2).

**Fig. 5:**
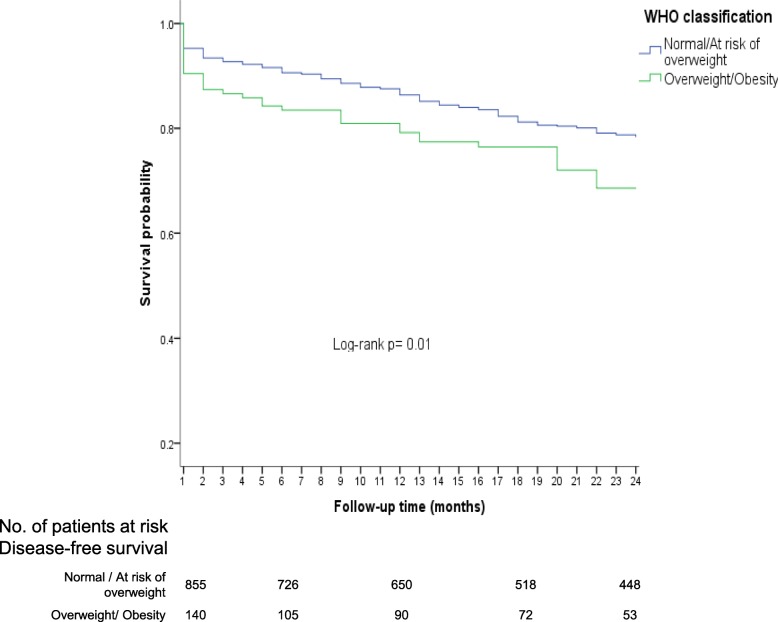
Overall survival up to 24 months after diagnosis according to WHO nutritional classification

Notably, overweight and obese patients had a lower OS (68 and 70%, respectively) in comparison with normal weight patients (OS = 80%). In the subgroup at risk of overweight, OS was 75%, slightly superior to that observed in patients with overweight or obesity (Log-rank *p* = 0.04). When categorized together as the reference group patients with normal weight and at risk of overweight, and patients with overweight and obesity as another group, differences in OS up to 24 months became more noticeable (Log Rank; *p* = 0.01) (Fig. 5).

Overweight was associated with an increased risk of early mortality (HR = 1.5; 95% CI: 1.0–2.3) but for obesity an imprecision was observed (HR = 1.5; 95% CI: 0.8–2.7). For this reason, when considering the reference group (normal/at risk of overweight) for further comparison with the overweight/obesity group, a high risk of early mortality was detected (HR = 1.4, 95% CI: 1.0–2.0).

In a separate analysis, the association between overweight/obesity and early mortality also was assessed stratifying by NCI risk classification. Similar results were observed in the subgroup of standard-risk (HR = 1.5, 95% CI: 0.7–2.9) and high-risk patients (HR = 1.5, 95% CI: 0.9–2.3).

Additionally, by age strata, the risk of early mortality in the group of obese patients was higher in the age group ≥10 years (HR = 2.0, 95% CI: 1.2–3.3) than in the group of 1–9.9 years (HR = 1.2, 95% CI: 0.7–2.2). It is important to highlight that when ALL infants (< 1 year) were excluded from the analyses, the risks did not change.

## Discussion

Mexico is one of the few countries around the globe where mortality in children with ALL has not been reduced, on the contrary, it has been observed a trend to increase in recent years [1, 2]. In addition, it is the country of Latin America with the lowest survival only surpassed by Ecuador [28]. In previous studies, it has been reported that there are differences in the clinical characteristics at diagnosis of Mexican patients with ALL in comparison to children from other populations where survival rates are better. For instance, almost 50% of ALL Mexican children are classified as having high risk of relapse according to NCI criteria, and in low proportion of children is detected the *ETV6-RUNX1* (7%), a gene rearrangement associated with a favorable prognosis [29]. On the other hand, in developed countries, only one third of patients are classified as having a high risk of relapse at the time of diagnosis and in 22% *ETV6-RUNX1* is detected [30].

In the present study, overweight and obesity at the time of diagnosis were predictors of early mortality in Mexican children with ALL.

To the best of our knowledge, the present research is the first to explore the association between overweight and obesity with early mortality and relapse risk in Mexican children with ALL. A frequency of 19.9% for early mortality during the first 24 months after diagnosis confirmation was noted; particularly, the excess mortality was clustered in the first 2 months from diagnosis. This is high, considering the reported for the same follow-up period in ALL children from developed countries (~ 6.8%) [13].

Actually, a high prevalence of overweight and obesity has been reported in patients at ALL diagnosis confirmation and in survivors from this disease, in comparison with other types of childhood cancer [31]. Specifically, ALL children from a Hispanic ethnicity display the highest rates of overweight and obesity during treatment [32].

In other populations, as similar to the findings of the present research, the presence of overweight and obesity have been associated with dismal outcomes and lower survival rates.

Orgel et al [13] in a meta-analysis of 11 studies reported a high risk of mortality for overweight/obese children (RR = 1.3, 95% CI: 1.1–1.6). In another meta-analysis by Amankwah et al [33] an association between obesity at the time of diagnosis and a high risk of dying within the first 5-years of follow-up also had been noted (HR = 1.3, 95% CI: 1.2–1.5). These findings were similar to the reported by other researchers such as Calle et al [20], and Ethier et al [18]. On the contrary, an imprecise association between obesity and low survival rates has been documented in a cohort of predominantly Hispanic children with ALL (HR = 1.4, 95% CI = 0.69–2.87) [17].

In the current study, both the CDC 2000 and the WHO growth charts were used to classify nutritional status by BMI at diagnosis. It has been reported that BMI correlates well with direct body fat measures (skinfold thickness, bioelectrical impedance, densitometry dual energy x-ray absorptiometry and other methods) [34]. Therefore, using both classifications makes the results obtained in the current research, valid and with greater possibilities for further comparison [13].

To carry on, frequencies for overweight/obesity using CDC (27.1%) and WHO (13%) were inferior to those reported for ALL children from other populations where different nutritional classifications were used, such as the United Kingdom (35%, IOTF) [35], Brazil (35.9%, WHO) [14] and Canada (33.2%, CDC) [18]. Nevertheless, the prevalence by CDC was similar to that reported for ALL children from Malaysia, China and India (24.5%, WHO) [36].

On the other hand, the frequency of early relapse in this study was 14.9%, lower than the previously reported in a tertiary Mexican public hospital by Jimenez-Hernández et al [10] (22.1%), similar to the referred by Antillón et al [37] in Guatemala (14.9%), but higher than the observed in developed countries (< 5%) [38]. The results of the present study about the association between early relapse risk for overweight or obesity at the time of diagnosis, were in the borderline of precision from a statistical perspective. In previous studies, the relationship between overweight and obesity and risk of relapse has been evaluated at least for a 5-year follow-up period and results have been diverse [14, 31, 39]. For instance, in the meta-analysis by Amankwah et al [33] one study reported a low risk (HR = 0.73, 95% CI = 0.59–0.91), while another reported an elevated one (HR = 1.29, 95% CI = 1.02–1.56) for obese children. In other study conducted by Gelelete et al [14], they reported a high risk (HR = 1.3, 95% CI: 1.0–1.6) for relapse at a 5-years follow-up in overweight/obese ALL children. On the other hand, Aplenc *et al* [39] studied 768 ALL children and found a lower risk of relapse in obese patients (HR = 0.7, 95% CI: 0.6–0.9), in comparison to normal weight children at the time of diagnosis.

One possible reason for the imprecision in risk estimation for early relapse in the present study, was a low proportion of relapse events during research. For this reason, it would be important to continue to follow-up this cohort at least for three more years in order to evaluate the association of these nutritional alterations with relapse rates in Mexican ALL children.

Currently, dose adjustments for chemotherapy drugs in leukemia children are based on the patient’s total body weight and/or body surface area. Up to date, there is no evidence about which is the best way for chemotherapy dosage and intensity adjustment in overweight and obese patients. It is well recognized that the body weight do not correlate with body fat percentage which is elevated in overweight and obese patients as well [40]. This could lead to the patient being given supra-therapeutic or subtherapeutic doses of the medication and thereby increasing the risk of toxicity and death, or relapse [40].

Taking into account the results obtained in the present work and previously reported on the association between overweight and obesity with the high risk of dying, the overweight/obese children should be considered as a subgroup of patients that require a closer monitoring given the high possibility for developing severe complications associated with an increased risk of death. Therefore, it is relevant to further explore which other factors are contributing to increase the risk of dying in children with these conditions. Noteworthy, to increase the survival rates in Mexican children with ALL also requires to homogenize the chemotherapy treatment and supportive care protocols, as in populations with the highest survival rates [41, 42].

## Conclusions

Overweight and obesity were independent predictors of early mortality in Mexican children with ALL. A closer monitoring of these children would increase their survival. Importantly, further research is required for a deeper comprehension of the biological mechanisms by which overweight and obesity are involved in the association between treatment resistance and toxicity.

## Additional file

Additional file 1: **Table S1.** Comparison of overweight and obesity prevalence at diagnosis using CDC and WHO in ALL children. (DOCX 13 kb)

## Data Availability

The datasets generated and/or analyzed during the current study are not publicly available due to the fact that we are continually generating information, but such datasets are available from the corresponding author upon reasonable request.

## Abbreviations

ALL: Acute lymphoblastic leukemia
BMI: Body mass index
CDC: Centers for Disease Control and Prevention
CI: Confidence intervals
CNS: Central nervous system
CR: Complete remission
CSF: Cerebrospinal fluid
DFS: Disease free-survival
HR: Hazard ratios
MIGICCL: Mexican Interinstitutional Group for the Identification of the Causes of Childhood Leukemia
NCI: National Cancer Institute
OS: Overall survival
RR: Relative risks
SES: Socioeconomic status
WBC: White blood cell
WHO: World Health Organization
