# Supplementary material for: Overweight and obesity as predictors of early mortality in Mexican children with acute lymphoblastic leukemia: a multicenter cohort study
Source: BMC Cancer. 2019 Jul 18;19:708. doi: 10.1186/s12885-019-5878-8 (PMC6639907; doi:10.1186/s12885-019-5878-8)
Supplement: Supplementary file 1 — Table S1. Comparison of overweight and obesity prevalence at diagnosis using CDC and WHO in ALL children. (DOCX 13 kb) [file 12885_2019_5878_MOESM1_ESM.docx]

**Additional Files**

Additional File 1: Table S1. Comparison of overweight and obesity prevalence at diagnosis using CDC and WHO in ALL children

| **Additional File 1: Table S1. Comparison of overweight and obesity prevalence at diagnosis using CDC and WHO in ALL children** | | | | |
| --- | --- | --- | --- | --- |
| **WHO** | **CDC 2000** | | | **Total** |
|  | **Normal (%)** | **Overweight (%)** | **Obesity (%)** |  |
| Normal | 521 (90.1) | 2 (1.6) | 4 (2.9) | 527 |
| At risk of overweight | 55 (9.5) | 122 (94.6) | 7 (5.0) | 189 |
| Overweight | 2 (0.3) | 5 (3.9) | 85 (61.2) | 92 |
| Obesity | --- | --- | 43 (30.9) | 43 |
| Total | 578 | 129 | 139 | 846 |
